# Integrated Analysis Identifies Four Genes as Novel Diagnostic Biomarkers Which Correlate with Immune Infiltration in Preeclampsia

**DOI:** 10.1155/2022/2373694

**Published:** 2022-04-28

**Authors:** Mu-yi Yang, Ming-hui Ji, Tian Shen, Lei Lei

**Affiliations:** ^1^Department of Obstetrics, Nanjing First Hospital, Nanjing Medical University, Nanjing, Jiangsu, China; ^2^School of Nursing, Nanjing Medical University, Nanjing, Jiangsu, China; ^3^Department of Pathology, Nanjing First Hospital, Nanjing Medical University, Nanjing, Jiangsu, China

## Abstract

Preeclampsia remains a high cause of incidence and death for mothers and fetuses in developing nations. Preeclampsia has numerous clinical and biochemical markers that have been tested, but they have failed to provide a conclusive diagnosis in the different phases of the disease's progression. Herein, our team intended to determine potential diagnostic biomarkers for preeclampsia and analyzed associations with immune cells. Two microarray data from mankind's preeclampsia and control specimens were acquired from GSE75010 and GSE44711 datasets. Differentially expressed genes (DEGs) were identified between77 normal samples and 80 preeclampsia samples. Candidate biomarkers were discovered using the least absolute shrinkage and selection operator (LASSO) and the support vector machine recursive feature elimination (SVM-RFE) analysis. The expressions and diagnostic values of genes in preeclampsia were further demonstrated in the GSE44711 dataset (8 control samples and 8 preeclampsia samples). The correlation of critical genes with the proportion of immune cells was analyzed. We identified 20 DEGs in preeclampsia. Diseases enriched by DEGs were mainly related to preeclampsia, gestational diabetes, ovarian disease, female reproductive system disease, and endocrine system disease. COL17A1, FLT1, FSTL3, and SERPINA3 were identified as diagnostic genes of preeclampsia and validated in the GSE44711 datasets. Immune cell infiltration assays suggested that COL17A1, FLT1, FSTL3, and SERPINA3 were related to several immune cells. Overall, we identified four critical diagnostic genes in preeclampsia. Furthermore, more well-designed research studies with larger cohorts were warranted to confirm the value of the four genes for the diagnosis and outcome of preeclampsia patients.

## 1. Introduction

Two percent to eight percent of all pregnant females have the complication of preeclampsia, which occurs when high blood pressure and proteinuria develop suddenly and without warning during pregnancy [1]. It has long-term effects on vascular and kidney health, as well as long-term disturbances of kidney and systemic physiology [2]. Although maternal mortality is especially high in developing countries, preeclampsia and its complications are one of the top four causes of maternal death even in developed societies [3, 4]. In addition, although some clinical and biochemical preeclampsia markers have been studied, they have been shown to be ineffectual in delivering a definitive diagnosis throughout the different phases of the disease's progression [5, 6]. Therefore, it is quite necessary to discover novel biomarkers which may provide more powerful and reliable diagnostic information for preeclampsia management.

Generally, in the absence of a definitive cause, preeclampsia is thought to be the result of a complex interplay between a number of maternal genes, environment factors (contamination, corpulence), and/or a deregulated immunoresponse on behalf of the mother in response to the paternal component of the fetal genotype [7, 8]. Several candidate biomarkers that have been associated with preeclampsia are implicated in placentation, regulation of blood pressure, inflammation, vascular formation, and function of endothelial cells [9, 10]. These markers included TLR4, LEP, INHA, Apo E, HIF-1a, HO-2, sFlt-1, PlGF, and EGF [11–13]. However, the sensitive biomarkers for prognosis and diagnosis were limited.

More and more interest has been aroused in terms of determining gene expression profiles on the foundation of gene chips and high-throughput sequencing for diagnoses and risk forecasts of substantial multifactor disorders, like preeclampsia [14–16]. Herein, our team intended to determine diagnostic biomarkers in preeclampsia based on machine learning.

## 2. Materials and Methods

### 2.1. Downloading and Preprocessing of Data

Firstly, our team acquired a set of preeclampsia chip data, GSE75010 and GSE44711, from the GEO datasets. GSE75010 datasets included 77 preeclampsia samples and 80 normal samples. GSE44711 included 8 preeclampsia samples and 8 normal samples. The data were processed via the following: (1) the multiple probes were mapped to the same gene, (2) the null probes were removed, (3) the probes were first mapped to the gene, and (4) the acquired dataset was log2-transformed quantile-normalized signal strength.

### 2.2. DEGs in Preeclampsia

Package limma in R was used to study data on gene expression differences between preeclampsia samples and normal samples, using thresholds of ∣log2 fold change (FC) | >0.8 and a modified *P* value of 0.05 or less.

### 2.3. Functional Enrichment Analysis of the DEGs

Then, GO enrichment and KEGG pathway enrichment analyses were completed to find the major biological attributes of DEGs. Adjusted *P* < 0.05 had significance in statistics. The “ggplot2” and “GOplot” packages in R were used to create the visual GO enrichment maps generated by the annotation analysis. Using R's “clusterProfiler” and “DOSE” packages, we ran enrichment analyses for disease ontology (DO) terms on DEGs [17].

### 2.4. Candidate Diagnostic Biomarker Screening

Preeclampsia diagnostic markers were classified using the least absolute shrinkage and selection operator (LASSO) method and support vector machine (SVM) arithmetic. glmnet's LASSO package was used, with the response type set to binomial and alpha set to 1. In addition, as a surveillant machine learning approach to support vectors, the support vector machine recursive feature elimination (SVM-RFE) identified the optimal variates via the deletion of the SVM-produced eigenvectors [18]. For the categorization analyses of the screened markers in the preeclampsia diagnoses, the SVM classifier from the R package e1071 was utilized.

### 2.5. Diagnostic Value of the Critical Genes in Preeclampsia

Using mRNA expression data from 77 normal samples and 80 preeclampsia samples, we created a ROC curve to investigate the predictive usefulness of the suggested biomarkers. Preeclampsia and control samples were separated using ROC assays, which were further validated in the GSE44711 datasets which included 8 control samples and 8 preeclampsia samples.

### 2.6. Immune Analysis

In each preeclampsia sample, CIBERSORT (http://cibersortx.stanford.edu) was utilized to identify the relative proportions of 22 invading immunocyte types. Every specimen's immunological scores were calculated using the ESTIMATE algorithm.

### 2.7. Analysis of Genes Identified and Immune Cells Infiltrated

The association of diagnostic genes with the amount of infiltration immunocytes was determined by the use of Spearman's rank correlative analyses in the R program. The ggplot2 package's chart approach was used to display the resulting correlations.

### 2.8. Statistical Analysis

The entire statistical analysis was completed via R program 4.0.2. *t*-tests and Wilcoxon rank-sum tests were used in the analysis of quantitative variables, depending on the type of data. The relationships between the expressions of the diagnostic genes and infiltration immunocytes were studied via the utilization of Spearman's correlation. The *P* value less than 0.05 was considered statistically significant.

## 3. Results

### 3.1. Determination of DEGs in Preeclampsia

**Figure 1:**
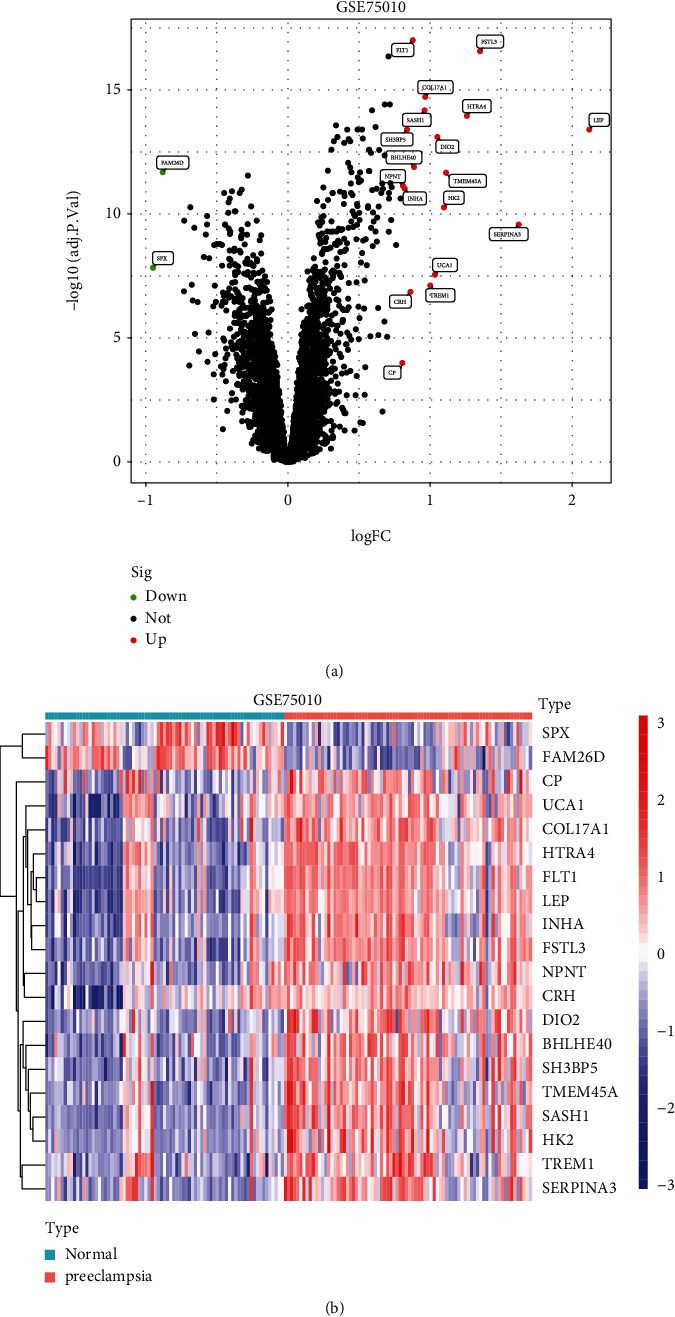
The dysregulated genes were shown in the (a) volcanic map and (b) heat map via analyzing GSE75010 datasets. 20 DEGs were identified between preeclampsia samples and normal samples.

To identify the DEGs in preeclampsia, our team studied GSE75010 datasets and screened 20 DEGs in preeclampsia, including 18 upregulated genes and 2 downregulated genes (Figures 1(a) and 1(b) and Table S1).

### 3.2. Functional Correlation Analysis

**Figure 2:**
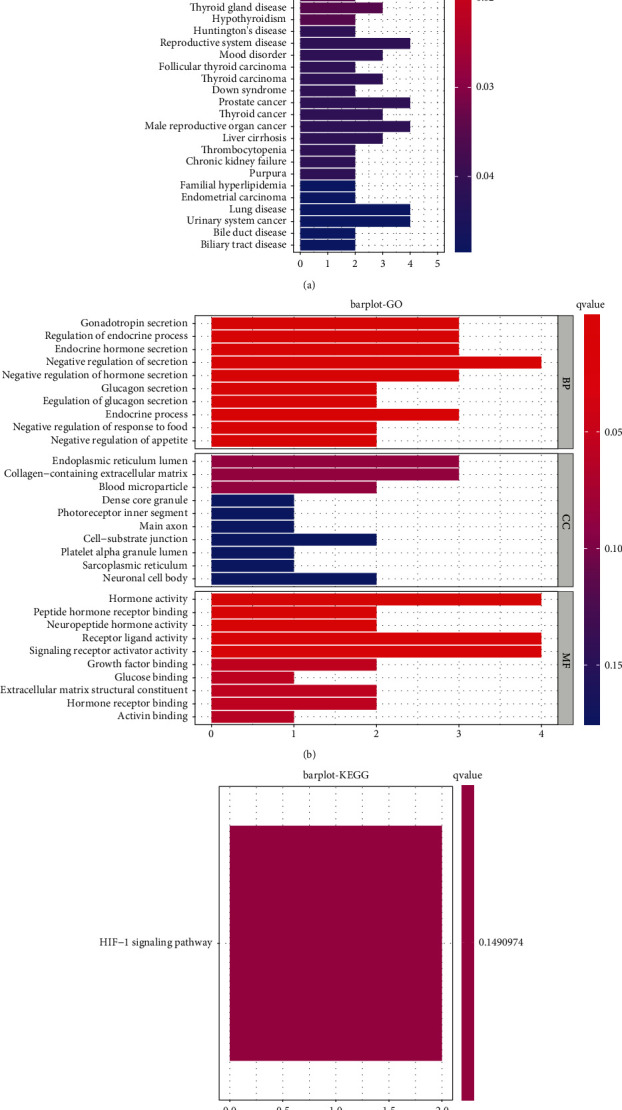
(a) DO, (b) GO, and (c) KEGG pathway analyses of 20 DEG in preeclampsia.

DO pathway enrichment analysis was completed to explore the roles of DEGs. The outcomes revealed that DEG-enriched illnesses were predominantly related to preeclampsia, gestational diabetes, ovarian disease, female reproductive system disease, and endocrine system disease (Figure 2(a) and Table S2). The results of GO analyses revealed that DEGs were mainly enriched in gonadotropin secretion, regulation of the endocrine process, endocrine hormone secretion, the endoplasmic reticulum lumen, extracellular matrix blood microparticles, dense core granules, hormonal activities, peptide hormonal acceptor binding, and neural peptide hormonal activities (Figure 2(b) and Table S3). KEGG assays revealed that DEGs were mainly enriched in the HIF-1 signaling pathway (Figure 2(c)).

### 3.3. Determination and Verification of Diagnostic Genes in Preeclampsia

**Figure 3:**
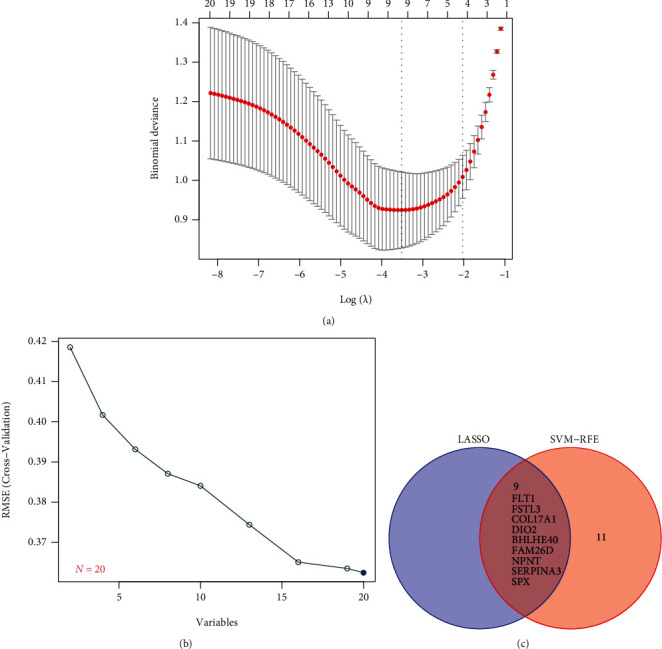
Selection procedure of diagnostic markers for preeclampsia diagnoses. (a) Tuning feature selection in the LASSO model. (b) An illustration of biological marker screening through the SVM-RFE arithmetic. (c) Venn graph presenting 4 diagnostic biomarkers shared by the LASSO and SVM-RFE arithmetic methods.

**Figure 4:**
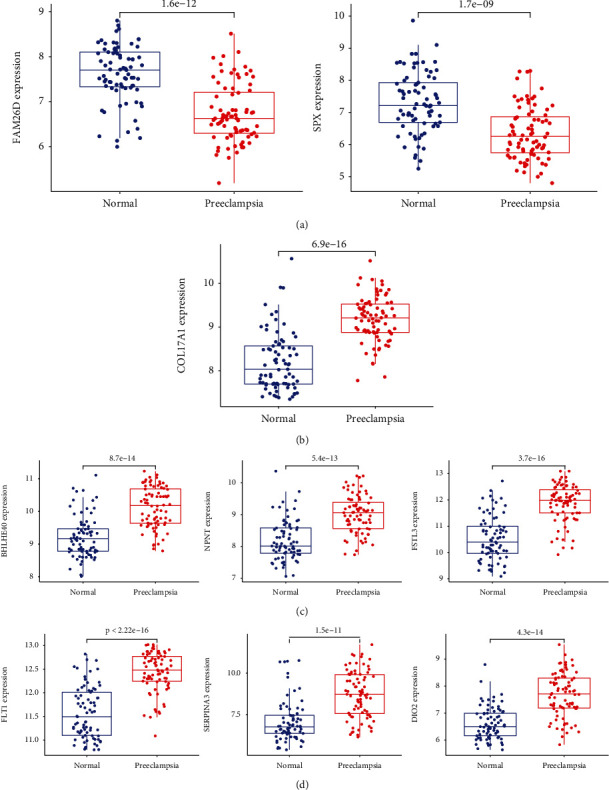
The expression pattern of the 9 critical genes in preeclampsia. (a) FAM26D and SPX were lowly expressed in preeclampsia. (b–d) FLT1, FSTL3, COL17A1, DIO2, BHLHE40, NPNT, and SERPINA3 were highly expressed in preeclampsia.

**Figure 5:**
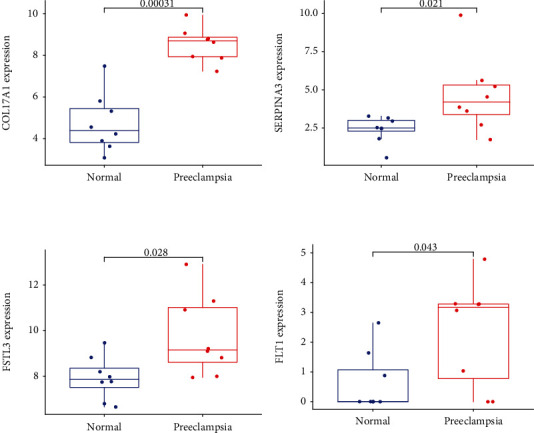
The expression pattern of the 9 critical genes was further demonstrated in GSE44711 datasets.

**Figure 6:**
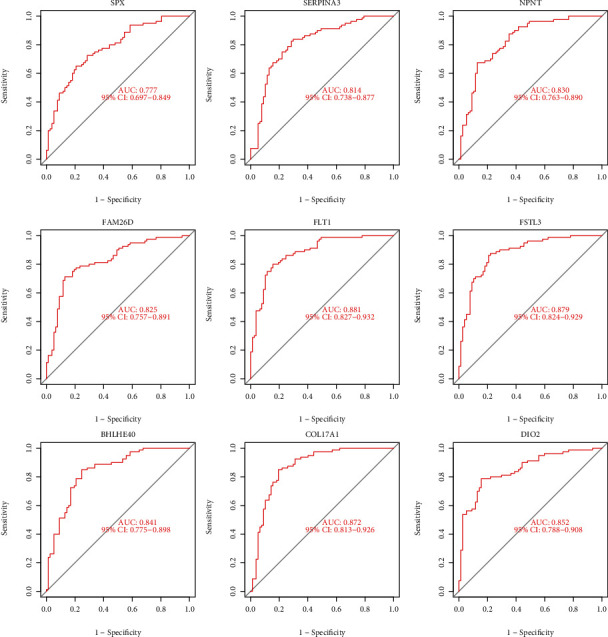
The diagnostic value of the 9 critical genes was studied using ROC assays in GSE75010.

**Figure 7:**
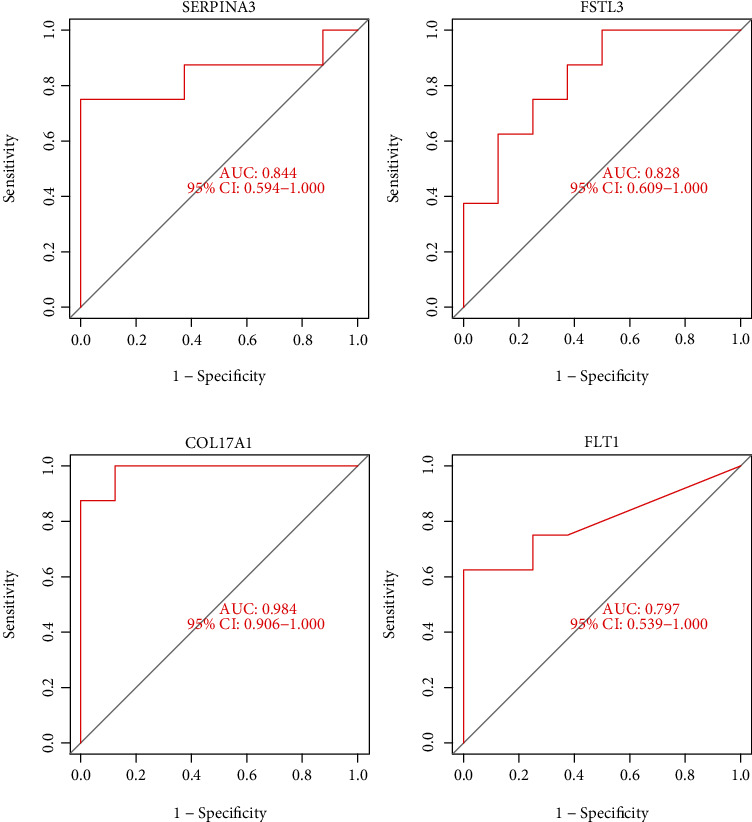
Among the 9 critical genes, four genes (COL17A1, FLT1, FSTL3, and SERPINA3) were further demonstrated to be diagnostic genes in GSE44711.

Two diverse arithmetic methods were employed to select underlying markers. The DEGs were identified via the LASSO regressive arithmetic, leading to the determination of 9 genes as diagnostic markers for preeclampsia (Figure 3(a)). A subgroup of 5 characteristics among the DEGs was identified via the SVM-RFE arithmetic (Figure 3(b)). The 9 overlapped genes (FLT1, FSTL3, COL17A1, DIO2, BHLHE40, FAM26D, NPNT, SERPINA3, and SPX) between both arithmetic methods were eventually acquired (Figure 3(c)). The expression of FAM26D and SPX was distinctly downregulated in preeclampsia (Figure 4(a)), and the expression of FLT1, FSTL3, COL17A1, DIO2, BHLHE40, NPNT, and SERPINA3 was distinctly upregulated in preeclampsia (Figures 4(b)–4(d)). In addition, to produce more precise and dependable outcomes, the GSE44711 dataset was employed to validate the expression level of the 9 characteristics. The distinct upregulation of COL17A1, SERPINA3, FSTL3, and FLT1 in preeclampsia was further demonstrated (Figure 5). The diagnostic value of nine overlapping genes was shown by the use of ROC assays (Figure 6). Moreover, we further determined the diagnostic value of COL17A1, SERPINA3, FSTL3, and FLT1 in GSE44711 datasets, and the results are shown in Figure 7.

### 3.4. Correlation between the Four Critical Genes and the Immune Infiltration Level in Preeclampsia

**Figure 8:**
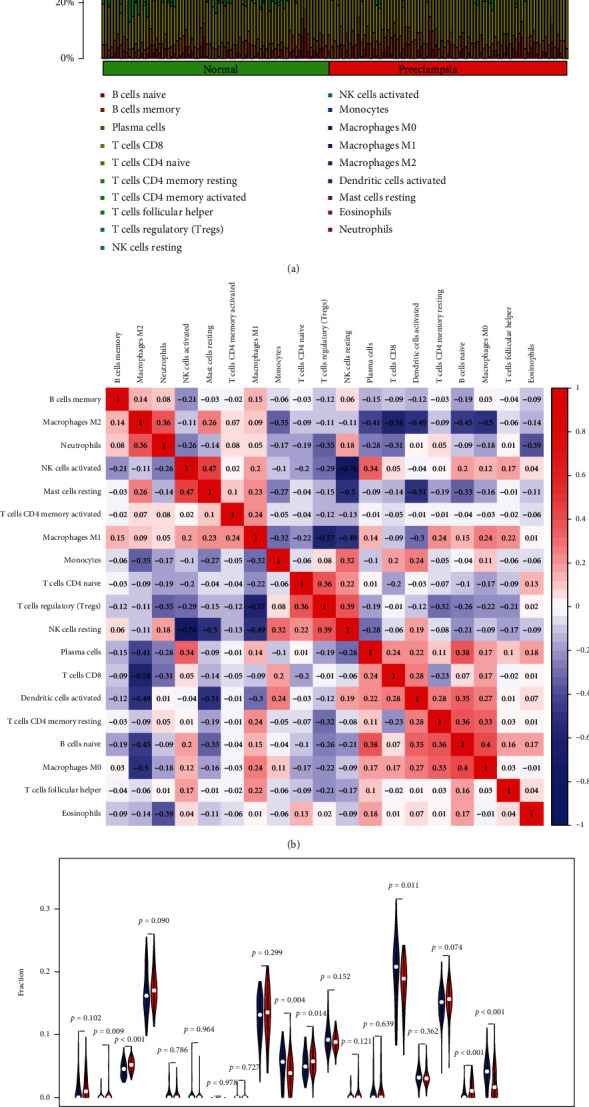
Immune cell profile in preeclampsia samples and correlation analysis. (a) The amounts of immunocytes in every preeclampsia specimen are indicated with diverse colors. (b) Correlative matrix for the entire 22 immunocyte amounts. (c) Contrast of 22 immunocyte subtypes between preeclampsia samples and normal tissues.

**Figure 9:**
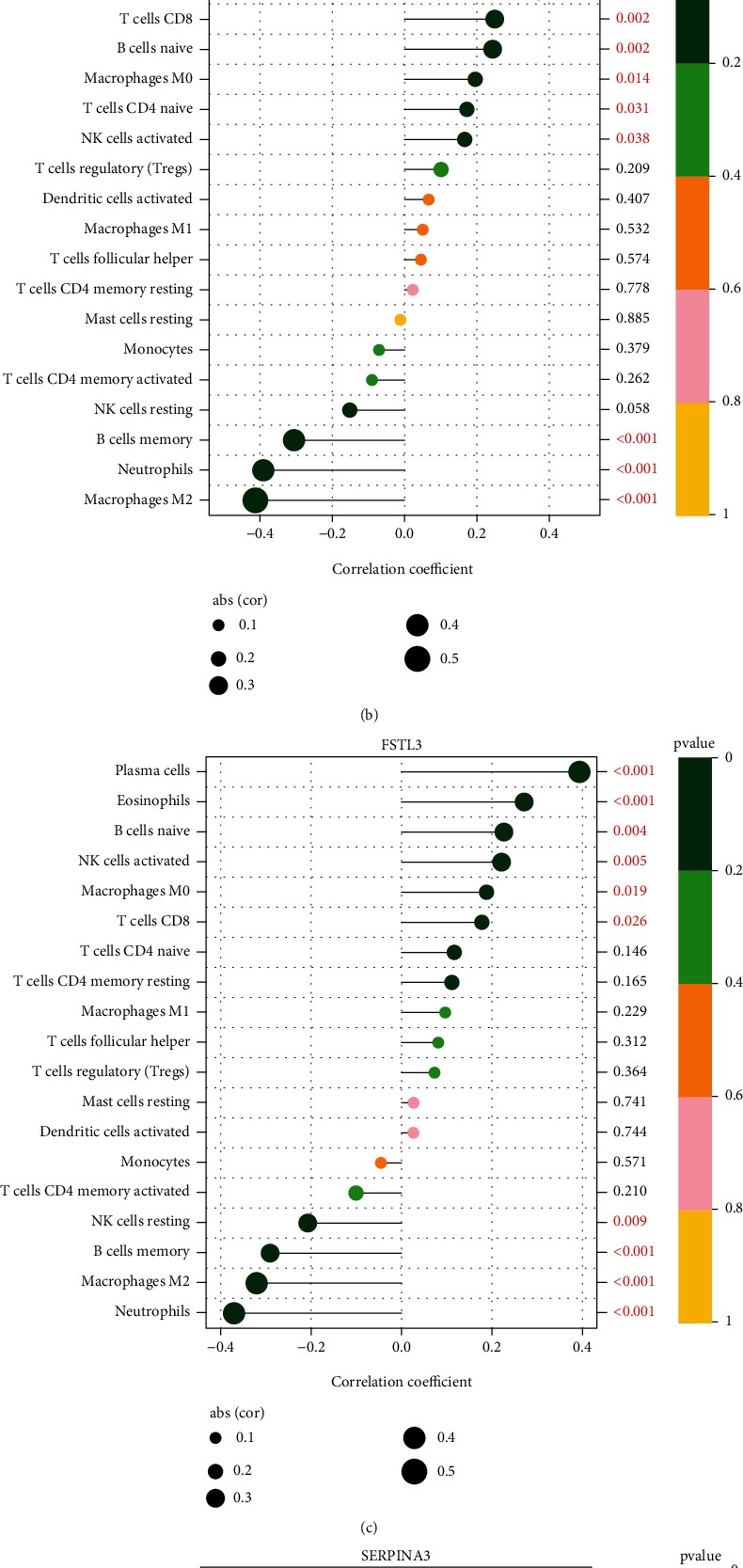
Correlation between (a) COL17A1, (b) FLT1, (c) FSTL3, and (d) SERPINA3 and infiltrating immune cells in preeclampsia.

For the purpose of determining the proportion of infiltrating immune subsets, the CIBERSORT method was used in conjunction with 21 different immune cell profiles built from samples of renal fibrosis in order to determine the relationship between the ISG20 and SERPINA expression and the immune microenvironment (Figures 8(a) and 8(b)). Several immune cells were observed to exhibit a dysregulated level in preeclampsia samples compared with normal samples (Figure 8(c)). We further explored the relationship between the expression of COL17A1, SERPINA3, FSTL3, and FLT1 and the immune infiltration level. As shown in Figure 9(a) and Figure S1, COL17A1 was positively correlated with plasma cells, eosinophils, B cells naïve, macrophages M0, NK cells stimulated, and T cells CD8 and related to NK cells resting in a negative way, including B cells memory, macrophages M2, and neutrophils. FLT1 was positively correlated with plasma cells, eosinophils, T cells CD8, B cells naïve, macrophages M0, T cells CD4 naïve, and NK cells stimulated and related to B cells memory in a negative way, including neutrophils and macrophages M2 (Figure 9(b) and Figure S2). FSTL3 was positively correlated with plasma cells, eosinophils, B cells naïve, NK cells stimulated, macrophages M0, and T cells CD8 and related to NK cells resting in a negative way, including B cells memory, macrophages M2, and neutrophils (Figure 9(c) and Figure S3). Moreover, SERPINA3 was related to plasma cells in a positive way, including eosinophils and NK cells activated, and related to macrophages M2 in a negative way, including NK cells resting, monocytes, B cells memory, and neutrophils (Figure 9(d) and Figure S4).

## 4. Discussion

Research on the pathogenesis of preeclampsia has unveiled substantial pivotal molecule factors, like soluble endoglin (sEng), soluble FMS-like tyrosine kinase-1 (sFlt-1), placental growth factor (PlGF), and vascular endothelial growth factor (VEGF) [19–21]. Vascular growth factors VEGF and PlGF are critical during embryonic development, while sFlt-1 and sEng exhibit antiangiogenic properties. Mounting studies have demonstrated that an imbalance between the above genes is related to the occurrence of the disease [22, 23]. The effects of those factors on the etiopathogenesis of the disease offer a chance for them to be utilized as underlying markers. However, more sensitive biomarkers were needed based on different methods. In this study, we analyzed GSE75010 datasets and identified 20 DEGs between preeclampsia samples and normal samples, including FLT1, FSTL3, COL17A1, SASH1, HTRA4, SH3BP5, LEP, DIO2, BHLHE40, FAM26D, TMEM45A, NPNT, INHA, HK2, SERPINA3, SPX, UCA1, TREM1, CRH, and CP. Interestingly, DO pathway enrichment analyses based on the above genes revealed that DEG-enriched illnesses were predominantly related to preeclampsia, gestational diabetes, ovarian disease, and female reproductive system disease, highlighting their involvement in the progression of preeclampsia. Our findings suggested the above 20 genes may be new promising target genes.

As a machine learning approach on the foundation of SVM, SVM-RFE is applied to observe the optimal variates via the deletion of SVM-produced feature vectors [24]. When the classification error is the lowest, the variable LASSO logistic regressive method is utilized to determine it [25]. In this study, we used SVM-RFE and LASSO logistic regressive methods to screen the possible biomarkers in preeclampsia and identified nine abnormally expressed genes, including FLT1, FSTL3, COL17A1, DIO2, BHLHE40, FAM26D, NPNT, SERPINA3, and SPX. ROC assays confirmed their diagnostic value in distinguishing preeclampsia samples from nontumor samples. To further demonstrate the above results, we further analyzed GSE44711 datasets and confirmed two overlapping genes in GSE44711 and GSE75010, including COL17A1, FLT1, FSTL3, and SERPINA3. A previous study has reported that an sFlt-1 : PlGF ratio of 38 or lower can be utilized to forecast the short-term absence of the disease in females whose syndromes are suspected [26]. Biron-Shental et al. reported that hypoxia culture remarkably reinforced the expressions of FSTL3 via trophoblasts [27]. The downregulated FSTL3 remarkably inhibited the proliferative, migratory, and invasive abilities and lipidic storage whereas elevated the programmed cell death of trophoblasts. The abnormal expressions of FSTL3 in the disease induced the aberrant function of trophoblasts, revealing its participation in the etiopathogenesis of the disease. These findings indicated the possible function of FLT1 and FSTL3 in the progression of preeclampsia. However, the expression and effects of COL17A1 and SERPINA3 have not been investigated. More studies should focus on the four genes.

The precise pathogenesis of preeclampsia remains elusive; nevertheless, endothelial aberrant function, improper angiogenetic activity, insufficient trophoblast invasive ability, and spiral uterine artery remodeling have been determined as pivotal contributing factors [28, 29]. The improper stimulation of the intrinsic immunosystem and following inflammatory events can induce placental aberrant function or unsatisfactory maternal blood vessels' adaptative ability and facilitate the progression of the disease [30, 31]. With the fast advancement of technologies, biological information has offered a potent method for selecting molecule biomarkers, and CIBERSORT kits have fostered the analyses of immunocyte infiltrative features of illnesses as well [32]. For the sake of investigating the effects of immunocyte infiltration on the disease, our team utilized CIBERSORT to finish an all-round assessment of the disease's immune infiltration. Our team discovered an elevated infiltration of B cells memory, plasma cells, NK cells activated, and eosinophils and a reduced infiltration of NK cells resting and neutrophils. Moreover, we observed that the four genes were associated with many immunocytes. The literature reveals that decidual NK cells facilitate trophoblast invasive ability via excreting chemotactic factors, and decidual macrophages act as antigen-presenting phagocytic cells, excreting cell factors and modulating the immune equilibrium between mothers and fetuses [33]. T cells and dendritic cells (DCs) are pivotal cells modulating the immune equilibrium [34, 35]. This demonstrates the significance of immunocyte infiltration in the etiopathogenesis and immunotyping of preeclampsia. Our findings together with previous findings suggested COL17A1, FLT1, FSTL3, and SERPINA3 may influence the immune function of preeclampsia patients.

This study also has some shortcomings. Firstly, our team acquired data merely from the GEO datasets, and the sample size was not quite large. More research studies with bigger sample sizes ought to complete to verify the discoveries herein. Secondly, although 4 critical genes were identified as potential biomarkers for preeclampsia immunotyping, no in vivo or in vitro studies were carried out, so this should be a focus in future work.

## 5. Conclusion

Our study identified COL17A1, FLT1, FSTL3, and SERPINA3 as novel diagnostic biomarkers for preeclampsia patients. In addition, their associations with immune cell infiltration may promote the development of immunotherapy in patients with preeclampsia.

## Data Availability

The data used to support the findings of this study are available from the corresponding author upon request.

## Supplementary Materials

Supplementary Materials Figure S1: correlation of the immune cell percentage with the expression of COL17A1 in preeclampsia. Figure S2: correlation of the immune cell percentage with the expression of FLT1 in preeclampsia. Figure S3: correlation of the immune cell percentage with the expression of FSTL3 in preeclampsia. Figure S4: correlation of the immune cell percentage with the expression of SERPINA3 in preeclampsia. Table S1: the abnormally expressed genes in preeclampsia. Table S2: the details of disease ontology enrichment analysis. Table S3: the details of gene ontology (GO) terms.
