# Supplementary material for: Integrated Analysis Identifies Four Genes as Novel Diagnostic Biomarkers Which Correlate with Immune Infiltration in Preeclampsia
Source: J Immunol Res. 2022 Apr 28;2022:2373694. doi: 10.1155/2022/2373694 (PMC9071854; doi:10.1155/2022/2373694)
Supplement: Supplementary Materials — Figure S1: correlation of the immune cell percentage with the expression of COL17A1 in preeclampsia. Figure S2: correlation of the immune cell percentage with the expression of FLT1 in preeclampsia. Figure S3: correlation of the immune cell percentage with the expression of FSTL3 in preeclampsia. Figure S4: correlation of the immune cell percentage with the expression of SERPINA3 in preeclampsia. Table S1: the abnormally expressed genes in preeclampsia. Table S2: the details of disease ontology enrichment analysis. Table S3: the details of gene ontology (GO) terms. [file 2373694.f1.zip › Table S1.docx]

**Table S1** The abnormally expressed genes in preeclampsia.

| id | logFC | AveExpr | t | P.Value | adj.P.Val | B |
| --- | --- | --- | --- | --- | --- | --- |
| FLT1 | 0.878449 | 12.00982 | 11.28774 | 4.29E-22 | 9.98E-18 | 39.40995 |
| FSTL3 | 1.351484 | 11.22605 | 11.01704 | 2.37E-21 | 2.76E-17 | 37.75376 |
| COL17A1 | 0.966245 | 8.691949 | 10.23135 | 3.28E-19 | 1.91E-15 | 32.97628 |
| SASH1 | 0.961485 | 9.938823 | 9.93461 | 2.08E-18 | 6.67E-15 | 31.18789 |
| HTRA4 | 1.259204 | 11.38769 | 9.816646 | 4.32E-18 | 1.12E-14 | 30.48003 |
| SH3BP5 | 0.838121 | 9.322049 | 9.52116 | 2.67E-17 | 3.99E-14 | 28.71563 |
| LEP | 2.120384 | 10.16282 | 9.516611 | 2.74E-17 | 3.99E-14 | 28.68857 |
| DIO2 | 1.051294 | 7.146265 | 9.386203 | 6.09E-17 | 7.92E-14 | 27.91432 |
| BHLHE40 | 0.887302 | 9.686411 | 8.841573 | 1.66E-15 | 1.29E-12 | 24.71413 |
| FAM26D | -0.88105 | 7.180234 | -8.72933 | 3.25E-15 | 2.10E-12 | 24.06209 |
| TMEM45A | 1.112329 | 8.423944 | 8.713063 | 3.58E-15 | 2.19E-12 | 23.9678 |
| NPNT | 0.809374 | 8.605388 | 8.475977 | 1.47E-14 | 7.43E-12 | 22.60084 |
| INHA | 0.821537 | 8.946406 | 8.417515 | 2.07E-14 | 9.66E-12 | 22.26585 |
| HK2 | 1.097858 | 8.454656 | 8.040752 | 1.89E-13 | 5.49E-11 | 20.1284 |
| SERPINA3 | 1.623422 | 7.904333 | 7.717949 | 1.21E-12 | 2.68E-10 | 18.32925 |
| SPX | -0.94962 | 6.822026 | -6.87852 | 1.30E-10 | 1.49E-08 | 13.81418 |
| UCA1 | 1.0345 | 10.67601 | 6.738262 | 2.76E-10 | 2.82E-08 | 13.08587 |
| TREM1 | 1.00165 | 9.358697 | 6.518294 | 8.88E-10 | 7.77E-08 | 11.96038 |
| CRH | 0.862013 | 11.01001 | 6.387726 | 1.76E-09 | 1.39E-07 | 11.30239 |
| CP | 0.805002 | 6.892491 | 4.762051 | 4.27E-06 | 0.000102 | 3.844973 |
