# Supplementary material for: Integrated Analysis Identifies Four Genes as Novel Diagnostic Biomarkers Which Correlate with Immune Infiltration in Preeclampsia
Source: J Immunol Res. 2022 Apr 28;2022:2373694. doi: 10.1155/2022/2373694 (PMC9071854; doi:10.1155/2022/2373694)
Supplement: Supplementary Materials — Figure S1: correlation of the immune cell percentage with the expression of COL17A1 in preeclampsia. Figure S2: correlation of the immune cell percentage with the expression of FLT1 in preeclampsia. Figure S3: correlation of the immune cell percentage with the expression of FSTL3 in preeclampsia. Figure S4: correlation of the immune cell percentage with the expression of SERPINA3 in preeclampsia. Table S1: the abnormally expressed genes in preeclampsia. Table S2: the details of disease ontology enrichment analysis. Table S3: the details of gene ontology (GO) terms. [file 2373694.f1.zip › Table S2.docx]

Table S2 The details of Disease ontology enrichment analysis.

| ID | Description |  | BgRatio | pvalue | p.adjust | qvalue | geneID | Count |
| --- | --- | --- | --- | --- | --- | --- | --- | --- |
| DOID:10591 | pre-eclampsia |  | 278/8007 | 7.53E-05 | 0.013931 | 0.008354 | FLT1/LEP/INHA/CRH/CP | 5 |
| DOID:11714 | gestational diabetes |  | 60/8007 | 0.000137 | 0.013931 | 0.008354 | FLT1/FSTL3/LEP | 3 |
| DOID:1100 | ovarian disease |  | 64/8007 | 0.000167 | 0.013931 | 0.008354 | FLT1/LEP/INHA | 3 |
| DOID:229 | female reproductive system disease |  | 193/8007 | 0.000271 | 0.017005 | 0.010198 | FLT1/FSTL3/LEP/INHA | 4 |
| DOID:28 | endocrine system disease |  | 399/8007 | 0.000413 | 0.020716 | 0.012423 | FLT1/LEP/DIO2/INHA/TREM1 | 5 |
| DOID:811 | lipodystrophy |  | 34/8007 | 0.001543 | 0.058767 | 0.035243 | LEP/DIO2 | 2 |
| DOID:3973 | thyroid medullary carcinoma |  | 38/8007 | 0.001925 | 0.058767 | 0.035243 | FLT1/DIO2 | 2 |
| DOID:2021 | placenta cancer |  | 41/8007 | 0.002239 | 0.058767 | 0.035243 | LEP/DIO2 | 2 |
| DOID:3594 | choriocarcinoma |  | 41/8007 | 0.002239 | 0.058767 | 0.035243 | LEP/DIO2 | 2 |
| DOID:50 | thyroid gland disease |  | 159/8007 | 0.002381 | 0.058767 | 0.035243 | FLT1/LEP/DIO2 | 3 |
| DOID:1459 | hypothyroidism |  | 44/8007 | 0.002575 | 0.058767 | 0.035243 | LEP/DIO2 | 2 |
| DOID:12858 | Huntington's disease |  | 52/8007 | 0.003581 | 0.068872 | 0.041303 | LEP/CP | 2 |
| DOID:15 | reproductive system disease |  | 386/8007 | 0.003619 | 0.068872 | 0.041303 | FLT1/FSTL3/LEP/INHA | 4 |
| DOID:3324 | mood disorder |  | 189/8007 | 0.003889 | 0.068872 | 0.041303 | DIO2/BHLHE40/CRH | 3 |
| DOID:3962 | follicular thyroid carcinoma |  | 56/8007 | 0.004143 | 0.068872 | 0.041303 | FLT1/DIO2 | 2 |
| DOID:3963 | thyroid carcinoma |  | 200/8007 | 0.00456 | 0.068872 | 0.041303 | FLT1/LEP/DIO2 | 3 |
| DOID:14250 | Down syndrome |  | 61/8007 | 0.004898 | 0.068872 | 0.041303 | LEP/INHA | 2 |
| DOID:10283 | prostate cancer |  | 425/8007 | 0.005118 | 0.068872 | 0.041303 | FLT1/LEP/INHA/SERPINA3 | 4 |
| DOID:1781 | thyroid cancer |  | 213/8007 | 0.005439 | 0.068872 | 0.041303 | FLT1/LEP/DIO2 | 3 |
| DOID:3856 | male reproductive organ cancer |  | 435/8007 | 0.005561 | 0.068872 | 0.041303 | FLT1/LEP/INHA/SERPINA3 | 4 |
| DOID:5082 | liver cirrhosis |  | 220/8007 | 0.005952 | 0.068872 | 0.041303 | FLT1/LEP/SERPINA3 | 3 |
| DOID:1588 | thrombocytopenia |  | 68/8007 | 0.006055 | 0.068872 | 0.041303 | LEP/CP | 2 |
| DOID:784 | chronic kidney failure |  | 70/8007 | 0.006406 | 0.068872 | 0.041303 | LEP/CRH | 2 |
| DOID:3326 | purpura |  | 71/8007 | 0.006585 | 0.068872 | 0.041303 | LEP/CP | 2 |
| DOID:1168 | familial hyperlipidemia |  | 82/8007 | 0.008705 | 0.080757 | 0.04843 | LEP/CP | 2 |
| DOID:2871 | endometrial carcinoma |  | 82/8007 | 0.008705 | 0.080757 | 0.04843 | LEP/INHA | 2 |
| DOID:850 | lung disease |  | 499/8007 | 0.009033 | 0.080757 | 0.04843 | FLT1/LEP/INHA/TREM1 | 4 |
| DOID:3996 | urinary system cancer |  | 500/8007 | 0.009096 | 0.080757 | 0.04843 | FLT1/LEP/INHA/UCA1 | 4 |
| DOID:4138 | bile duct disease |  | 86/8007 | 0.009542 | 0.080757 | 0.04843 | TREM1/CP | 2 |
| DOID:9741 | biliary tract disease |  | 87/8007 | 0.009757 | 0.080757 | 0.04843 | TREM1/CP | 2 |
| DOID:289 | endometriosis |  | 88/8007 | 0.009974 | 0.080757 | 0.04843 | FSTL3/LEP | 2 |
| DOID:3146 | lipid metabolism disorder |  | 92/8007 | 0.010863 | 0.083177 | 0.049882 | LEP/CP | 2 |
| DOID:3717 | gastric adenocarcinoma |  | 94/8007 | 0.011321 | 0.083177 | 0.049882 | LEP/HK2 | 2 |
| DOID:9452 | fatty liver disease |  | 95/8007 | 0.011553 | 0.083177 | 0.049882 | FLT1/LEP | 2 |
| DOID:2218 | blood platelet disease |  | 96/8007 | 0.011787 | 0.083177 | 0.049882 | LEP/CP | 2 |
| DOID:1380 | endometrial cancer |  | 97/8007 | 0.012023 | 0.083177 | 0.049882 | LEP/INHA | 2 |
| DOID:363 | uterine cancer |  | 98/8007 | 0.012261 | 0.083177 | 0.049882 | LEP/INHA | 2 |
