# Supplementary material for: Integrated Analysis Identifies Four Genes as Novel Diagnostic Biomarkers Which Correlate with Immune Infiltration in Preeclampsia
Source: J Immunol Res. 2022 Apr 28;2022:2373694. doi: 10.1155/2022/2373694 (PMC9071854; doi:10.1155/2022/2373694)
Supplement: Supplementary Materials — Figure S1: correlation of the immune cell percentage with the expression of COL17A1 in preeclampsia. Figure S2: correlation of the immune cell percentage with the expression of FLT1 in preeclampsia. Figure S3: correlation of the immune cell percentage with the expression of FSTL3 in preeclampsia. Figure S4: correlation of the immune cell percentage with the expression of SERPINA3 in preeclampsia. Table S1: the abnormally expressed genes in preeclampsia. Table S2: the details of disease ontology enrichment analysis. Table S3: the details of gene ontology (GO) terms. [file 2373694.f1.zip › Table S3.docx]

**Table S3 The details of gene ontology (GO) terms.**

| ONTOLOGY | ID | Description | BgRatio | pvalue | p.adjust | qvalue | geneID | Count |
| --- | --- | --- | --- | --- | --- | --- | --- | --- |
| BP | GO:0032274 | gonadotropin secretion | 13/18862 | 2.07E-07 | 0.000175 | 0.000108 | LEP/INHA/CRH | 3 |
| BP | GO:0044060 | regulation of endocrine process | 34/18862 | 4.29E-06 | 0.001811 | 0.001119 | LEP/INHA/CRH | 3 |
| BP | GO:0060986 | endocrine hormone secretion | 45/18862 | 1.01E-05 | 0.002844 | 0.001757 | LEP/INHA/CRH | 3 |
| BP | GO:0051048 | negative regulation of secretion | 160/18862 | 1.39E-05 | 0.002938 | 0.001815 | LEP/INHA/SPX/CRH | 4 |
| BP | GO:0046888 | negative regulation of hormone secretion | 60/18862 | 2.41E-05 | 0.004079 | 0.00252 | LEP/INHA/CRH | 3 |
| BP | GO:0070091 | glucagon secretion | 10/18862 | 3.85E-05 | 0.004651 | 0.002874 | LEP/CRH | 2 |
| BP | GO:0070092 | regulation of glucagon secretion | 10/18862 | 3.85E-05 | 0.004651 | 0.002874 | LEP/CRH | 2 |
| BP | GO:0050886 | endocrine process | 78/18862 | 5.31E-05 | 0.004695 | 0.002901 | LEP/INHA/CRH | 3 |
| BP | GO:0032096 | negative regulation of response to food | 13/18862 | 6.67E-05 | 0.004695 | 0.002901 | LEP/SPX | 2 |
| BP | GO:0032099 | negative regulation of appetite | 13/18862 | 6.67E-05 | 0.004695 | 0.002901 | LEP/SPX | 2 |
| BP | GO:0032105 | negative regulation of response to extracellular stimulus | 13/18862 | 6.67E-05 | 0.004695 | 0.002901 | LEP/SPX | 2 |
| BP | GO:0032108 | negative regulation of response to nutrient levels | 13/18862 | 6.67E-05 | 0.004695 | 0.002901 | LEP/SPX | 2 |
| BP | GO:0022600 | digestive system process | 99/18862 | 0.000108 | 0.007025 | 0.004341 | LEP/SERPINA3/CRH | 3 |
| BP | GO:0032095 | regulation of response to food | 19/18862 | 0.000146 | 0.008793 | 0.005433 | LEP/SPX | 2 |
| BP | GO:0046887 | positive regulation of hormone secretion | 124/18862 | 0.000211 | 0.010827 | 0.006689 | LEP/INHA/CRH | 3 |
| BP | GO:0032098 | regulation of appetite | 23/18862 | 0.000215 | 0.010827 | 0.006689 | LEP/SPX | 2 |
| BP | GO:0045765 | regulation of angiogenesis | 335/18862 | 0.000246 | 0.010827 | 0.006689 | FLT1/SASH1/LEP/HK2 | 4 |
| BP | GO:1901342 | regulation of vasculature development | 341/18862 | 0.000263 | 0.010827 | 0.006689 | FLT1/SASH1/LEP/HK2 | 4 |
| BP | GO:0032104 | regulation of response to extracellular stimulus | 26/18862 | 0.000276 | 0.010827 | 0.006689 | LEP/SPX | 2 |
| BP | GO:0032107 | regulation of response to nutrient levels | 26/18862 | 0.000276 | 0.010827 | 0.006689 | LEP/SPX | 2 |
| BP | GO:0051953 | negative regulation of amine transport | 26/18862 | 0.000276 | 0.010827 | 0.006689 | LEP/CRH | 2 |
| BP | GO:0007586 | digestion | 138/18862 | 0.000288 | 0.010827 | 0.006689 | LEP/SERPINA3/CRH | 3 |
| BP | GO:1903531 | negative regulation of secretion by cell | 139/18862 | 0.000295 | 0.010827 | 0.006689 | LEP/INHA/CRH | 3 |
| BP | GO:1900745 | positive regulation of p38MAPK cascade | 29/18862 | 0.000344 | 0.012109 | 0.007482 | SASH1/LEP | 2 |
| BP | GO:0045766 | positive regulation of angiogenesis | 175/18862 | 0.000578 | 0.018035 | 0.011143 | FLT1/SASH1/HK2 | 3 |
| BP | GO:1904018 | positive regulation of vasculature development | 175/18862 | 0.000578 | 0.018035 | 0.011143 | FLT1/SASH1/HK2 | 3 |
| BP | GO:0032094 | response to food | 38/18862 | 0.000592 | 0.018035 | 0.011143 | LEP/SPX | 2 |
| BP | GO:0008217 | regulation of blood pressure | 177/18862 | 0.000598 | 0.018035 | 0.011143 | LEP/SPX/CRH | 3 |
| BP | GO:0044058 | regulation of digestive system process | 39/18862 | 0.000624 | 0.018187 | 0.011237 | LEP/CRH | 2 |
| BP | GO:0051051 | negative regulation of transport | 438/18862 | 0.000678 | 0.018806 | 0.01162 | LEP/INHA/SPX/CRH | 4 |
| BP | GO:0090278 | negative regulation of peptide hormone secretion | 41/18862 | 0.00069 | 0.018806 | 0.01162 | LEP/CRH | 2 |
| BP | GO:1900744 | regulation of p38MAPK cascade | 43/18862 | 0.000759 | 0.02004 | 0.012382 | SASH1/LEP | 2 |
| BP | GO:0050873 | brown fat cell differentiation | 46/18862 | 0.000868 | 0.022236 | 0.013739 | LEP/DIO2 | 2 |
| BP | GO:0002762 | negative regulation of myeloid leukocyte differentiation | 48/18862 | 0.000945 | 0.023031 | 0.01423 | FSTL3/INHA | 2 |
| BP | GO:0038066 | p38MAPK cascade | 49/18862 | 0.000985 | 0.023031 | 0.01423 | SASH1/LEP | 2 |
| BP | GO:0007623 | circadian rhythm | 212/18862 | 0.001008 | 0.023031 | 0.01423 | LEP/BHLHE40/CRH | 3 |
| BP | GO:0008406 | gonad development | 212/18862 | 0.001008 | 0.023031 | 0.01423 | FSTL3/LEP/INHA | 3 |
| BP | GO:0045137 | development of primary sexual characteristics | 217/18862 | 0.001079 | 0.023986 | 0.01482 | FSTL3/LEP/INHA | 3 |
| BP | GO:0042593 | glucose homeostasis | 229/18862 | 0.00126 | 0.026943 | 0.016648 | LEP/HK2/CRH | 3 |
| BP | GO:0033500 | carbohydrate homeostasis | 230/18862 | 0.001275 | 0.026943 | 0.016648 | LEP/HK2/CRH | 3 |
| BP | GO:0046324 | regulation of glucose import | 59/18862 | 0.001425 | 0.02937 | 0.018147 | LEP/HK2 | 2 |
| BP | GO:0090092 | regulation of transmembrane receptor protein serine/threonine kinase signaling pathway | 251/18862 | 0.001639 | 0.032971 | 0.020372 | FSTL3/HTRA4/INHA | 3 |
| BP | GO:0046883 | regulation of hormone secretion | 260/18862 | 0.001812 | 0.035578 | 0.021983 | LEP/INHA/CRH | 3 |
| BP | GO:0007548 | sex differentiation | 262/18862 | 0.001853 | 0.035578 | 0.021983 | FSTL3/LEP/INHA | 3 |
| BP | GO:1903532 | positive regulation of secretion by cell | 271/18862 | 0.00204 | 0.038305 | 0.023668 | LEP/INHA/CRH | 3 |
| BP | GO:0046323 | glucose import | 74/18862 | 0.002231 | 0.040975 | 0.025318 | LEP/HK2 | 2 |
| BP | GO:0002792 | negative regulation of peptide secretion | 76/18862 | 0.002351 | 0.042268 | 0.026116 | LEP/CRH | 2 |
| BP | GO:0010827 | regulation of glucose transmembrane transport | 77/18862 | 0.002412 | 0.042467 | 0.026239 | LEP/HK2 | 2 |
| BP | GO:0048511 | rhythmic process | 294/18862 | 0.002571 | 0.043876 | 0.02711 | LEP/BHLHE40/CRH | 3 |
| BP | GO:0051047 | positive regulation of secretion | 295/18862 | 0.002596 | 0.043876 | 0.02711 | LEP/INHA/CRH | 3 |
| BP | GO:0140353 | lipid export from cell | 81/18862 | 0.002665 | 0.044159 | 0.027284 | LEP/CRH | 2 |
| BP | GO:0046879 | hormone secretion | 302/18862 | 0.002775 | 0.045086 | 0.027857 | LEP/INHA/CRH | 3 |
| BP | GO:0051952 | regulation of amine transport | 86/18862 | 0.002998 | 0.047604 | 0.029413 | LEP/CRH | 2 |
| BP | GO:0009914 | hormone transport | 312/18862 | 0.003042 | 0.047604 | 0.029413 | LEP/INHA/CRH | 3 |
| BP | GO:0003073 | regulation of systemic arterial blood pressure | 90/18862 | 0.003278 | 0.049459 | 0.03056 | SPX/CRH | 2 |
| BP | GO:0014068 | positive regulation of phosphatidylinositol 3-kinase signaling | 90/18862 | 0.003278 | 0.049459 | 0.03056 | FLT1/LEP | 2 |
| BP | GO:0045638 | negative regulation of myeloid cell differentiation | 92/18862 | 0.003422 | 0.05073 | 0.031345 | FSTL3/INHA | 2 |
| BP | GO:0008585 | female gonad development | 93/18862 | 0.003495 | 0.050923 | 0.031464 | LEP/INHA | 2 |
| BP | GO:0015837 | amine transport | 94/18862 | 0.003569 | 0.051119 | 0.031585 | LEP/CRH | 2 |
| BP | GO:0046545 | development of primary female sexual characteristics | 97/18862 | 0.003796 | 0.052578 | 0.032487 | LEP/INHA | 2 |
| BP | GO:0120162 | positive regulation of cold-induced thermogenesis | 97/18862 | 0.003796 | 0.052578 | 0.032487 | LEP/DIO2 | 2 |
| BP | GO:1902106 | negative regulation of leukocyte differentiation | 101/18862 | 0.004107 | 0.055981 | 0.034589 | FSTL3/INHA | 2 |
| BP | GO:1903707 | negative regulation of hemopoiesis | 105/18862 | 0.004431 | 0.059109 | 0.036522 | FSTL3/INHA | 2 |
| BP | GO:0007178 | transmembrane receptor protein serine/threonine kinase signaling pathway | 358/18862 | 0.004477 | 0.059109 | 0.036522 | FSTL3/HTRA4/INHA | 3 |
| BP | GO:0002526 | acute inflammatory response | 107/18862 | 0.004597 | 0.059761 | 0.036925 | SERPINA3/TREM1 | 2 |
| BP | GO:1904659 | glucose transmembrane transport | 109/18862 | 0.004766 | 0.0598 | 0.036949 | LEP/HK2 | 2 |
| BP | GO:0018108 | peptidyl-tyrosine phosphorylation | 369/18862 | 0.004872 | 0.0598 | 0.036949 | FLT1/SH3BP5/LEP | 3 |
| BP | GO:0046660 | female sex differentiation | 111/18862 | 0.004938 | 0.0598 | 0.036949 | LEP/INHA | 2 |
| BP | GO:1903959 | regulation of anion transmembrane transport | 111/18862 | 0.004938 | 0.0598 | 0.036949 | LEP/HK2 | 2 |
| BP | GO:0018212 | peptidyl-tyrosine modification | 372/18862 | 0.004983 | 0.0598 | 0.036949 | FLT1/SH3BP5/LEP | 3 |
| BP | GO:0008645 | hexose transmembrane transport | 112/18862 | 0.005025 | 0.0598 | 0.036949 | LEP/HK2 | 2 |
| BP | GO:0015749 | monosaccharide transmembrane transport | 114/18862 | 0.005201 | 0.061027 | 0.037707 | LEP/HK2 | 2 |
| BP | GO:0032874 | positive regulation of stress-activated MAPK cascade | 116/18862 | 0.005379 | 0.061027 | 0.037707 | SASH1/LEP | 2 |
| BP | GO:0034219 | carbohydrate transmembrane transport | 116/18862 | 0.005379 | 0.061027 | 0.037707 | LEP/HK2 | 2 |
| BP | GO:0002761 | regulation of myeloid leukocyte differentiation | 118/18862 | 0.005561 | 0.061027 | 0.037707 | FSTL3/INHA | 2 |
| BP | GO:0042752 | regulation of circadian rhythm | 118/18862 | 0.005561 | 0.061027 | 0.037707 | BHLHE40/CRH | 2 |
| BP | GO:0070304 | positive regulation of stress-activated protein kinase signaling cascade | 118/18862 | 0.005561 | 0.061027 | 0.037707 | SASH1/LEP | 2 |
| BP | GO:0001503 | ossification | 401/18862 | 0.006138 | 0.06555 | 0.040502 | FSTL3/LEP/NPNT | 3 |
| BP | GO:0019216 | regulation of lipid metabolic process | 402/18862 | 0.00618 | 0.06555 | 0.040502 | FLT1/LEP/SERPINA3 | 3 |
| BP | GO:0048608 | reproductive structure development | 405/18862 | 0.006309 | 0.06555 | 0.040502 | FSTL3/LEP/INHA | 3 |
| BP | GO:0045471 | response to ethanol | 126/18862 | 0.006316 | 0.06555 | 0.040502 | LEP/CRH | 2 |
| BP | GO:0014066 | regulation of phosphatidylinositol 3-kinase signaling | 127/18862 | 0.006413 | 0.06555 | 0.040502 | FLT1/LEP | 2 |
| BP | GO:0061458 | reproductive system development | 408/18862 | 0.006439 | 0.06555 | 0.040502 | FSTL3/LEP/INHA | 3 |
| BP | GO:0071326 | cellular response to monosaccharide stimulus | 130/18862 | 0.006709 | 0.067494 | 0.041703 | LEP/CRH | 2 |
| BP | GO:0008584 | male gonad development | 134/18862 | 0.007114 | 0.069302 | 0.04282 | FSTL3/INHA | 2 |
| BP | GO:0090101 | negative regulation of transmembrane receptor protein serine/threonine kinase signaling pathway | 134/18862 | 0.007114 | 0.069302 | 0.04282 | FSTL3/HTRA4 | 2 |
| BP | GO:0045785 | positive regulation of cell adhesion | 425/18862 | 0.007206 | 0.069302 | 0.04282 | FSTL3/LEP/NPNT | 3 |
| BP | GO:0046546 | development of primary male sexual characteristics | 135/18862 | 0.007217 | 0.069302 | 0.04282 | FSTL3/INHA | 2 |
| BP | GO:0071322 | cellular response to carbohydrate stimulus | 138/18862 | 0.00753 | 0.071493 | 0.044174 | LEP/CRH | 2 |
| BP | GO:0106106 | cold-induced thermogenesis | 144/18862 | 0.008174 | 0.0759 | 0.046897 | LEP/DIO2 | 2 |
| BP | GO:0120161 | regulation of cold-induced thermogenesis | 144/18862 | 0.008174 | 0.0759 | 0.046897 | LEP/DIO2 | 2 |
| BP | GO:0001678 | cellular glucose homeostasis | 145/18862 | 0.008284 | 0.076082 | 0.047009 | HK2/CRH | 2 |
| BP | GO:0008643 | carbohydrate transport | 149/18862 | 0.008729 | 0.077459 | 0.04786 | LEP/HK2 | 2 |
| BP | GO:0006869 | lipid transport | 461/18862 | 0.009006 | 0.077459 | 0.04786 | LEP/SPX/CRH | 3 |
| BP | GO:0046661 | male sex differentiation | 153/18862 | 0.009185 | 0.077459 | 0.04786 | FSTL3/INHA | 2 |
| BP | GO:1990845 | adaptive thermogenesis | 153/18862 | 0.009185 | 0.077459 | 0.04786 | LEP/DIO2 | 2 |
| BP | GO:0014065 | phosphatidylinositol 3-kinase signaling | 154/18862 | 0.0093 | 0.077459 | 0.04786 | FLT1/LEP | 2 |
| BP | GO:0001820 | serotonin secretion | 10/18862 | 0.009504 | 0.077459 | 0.04786 | CRH | 1 |
| BP | GO:0008343 | adult feeding behavior | 10/18862 | 0.009504 | 0.077459 | 0.04786 | LEP | 1 |
| BP | GO:0032817 | regulation of natural killer cell proliferation | 10/18862 | 0.009504 | 0.077459 | 0.04786 | LEP | 1 |
| BP | GO:0035933 | glucocorticoid secretion | 10/18862 | 0.009504 | 0.077459 | 0.04786 | CRH | 1 |
| BP | GO:0072537 | fibroblast activation | 10/18862 | 0.009504 | 0.077459 | 0.04786 | LEP | 1 |
| BP | GO:1905709 | negative regulation of membrane permeability | 10/18862 | 0.009504 | 0.077459 | 0.04786 | HK2 | 1 |
| BP | GO:0032368 | regulation of lipid transport | 156/18862 | 0.009533 | 0.077459 | 0.04786 | LEP/CRH | 2 |
| BP | GO:0023061 | signal release | 475/18862 | 0.009772 | 0.077459 | 0.04786 | LEP/INHA/CRH | 3 |
| BP | GO:0002244 | hematopoietic progenitor cell differentiation | 161/18862 | 0.010128 | 0.077459 | 0.04786 | FLT1/FSTL3 | 2 |
| BP | GO:0001787 | natural killer cell proliferation | 11/18862 | 0.01045 | 0.077459 | 0.04786 | LEP | 1 |
| BP | GO:0006002 | fructose 6-phosphate metabolic process | 11/18862 | 0.01045 | 0.077459 | 0.04786 | HK2 | 1 |
| BP | GO:0010459 | negative regulation of heart rate | 11/18862 | 0.01045 | 0.077459 | 0.04786 | SPX | 1 |
| BP | GO:0033197 | response to vitamin E | 11/18862 | 0.01045 | 0.077459 | 0.04786 | LEP | 1 |
| BP | GO:0033210 | leptin-mediated signaling pathway | 11/18862 | 0.01045 | 0.077459 | 0.04786 | LEP | 1 |
| BP | GO:0042447 | hormone catabolic process | 11/18862 | 0.01045 | 0.077459 | 0.04786 | DIO2 | 1 |
| BP | GO:0042541 | hemoglobin biosynthetic process | 11/18862 | 0.01045 | 0.077459 | 0.04786 | INHA | 1 |
| BP | GO:1904478 | regulation of intestinal absorption | 11/18862 | 0.01045 | 0.077459 | 0.04786 | LEP | 1 |
| BP | GO:0001659 | temperature homeostasis | 171/18862 | 0.011365 | 0.078924 | 0.048765 | LEP/DIO2 | 2 |
| BP | GO:0003084 | positive regulation of systemic arterial blood pressure | 12/18862 | 0.011395 | 0.078924 | 0.048765 | SPX | 1 |
| BP | GO:0014831 | gastro-intestinal system smooth muscle contraction | 12/18862 | 0.011395 | 0.078924 | 0.048765 | SPX | 1 |
| BP | GO:0031581 | hemidesmosome assembly | 12/18862 | 0.011395 | 0.078924 | 0.048765 | COL17A1 | 1 |
| BP | GO:0032926 | negative regulation of activin receptor signaling pathway | 12/18862 | 0.011395 | 0.078924 | 0.048765 | FSTL3 | 1 |
| BP | GO:0035630 | bone mineralization involved in bone maturation | 12/18862 | 0.011395 | 0.078924 | 0.048765 | LEP | 1 |
| BP | GO:0051956 | negative regulation of amino acid transport | 12/18862 | 0.011395 | 0.078924 | 0.048765 | LEP | 1 |
| BP | GO:0060456 | positive regulation of digestive system process | 12/18862 | 0.011395 | 0.078924 | 0.048765 | CRH | 1 |
| BP | GO:0001936 | regulation of endothelial cell proliferation | 177/18862 | 0.012138 | 0.079591 | 0.049178 | FLT1/LEP | 2 |
| BP | GO:0007565 | female pregnancy | 177/18862 | 0.012138 | 0.079591 | 0.049178 | LEP/CRH | 2 |
| BP | GO:0050796 | regulation of insulin secretion | 178/18862 | 0.012269 | 0.079591 | 0.049178 | LEP/CRH | 2 |
| BP | GO:0007567 | parturition | 13/18862 | 0.012339 | 0.079591 | 0.049178 | CRH | 1 |
| BP | GO:0019336 | phenol-containing compound catabolic process | 13/18862 | 0.012339 | 0.079591 | 0.049178 | DIO2 | 1 |
| BP | GO:0033604 | negative regulation of catecholamine secretion | 13/18862 | 0.012339 | 0.079591 | 0.049178 | CRH | 1 |
| BP | GO:0045472 | response to ether | 13/18862 | 0.012339 | 0.079591 | 0.049178 | CRH | 1 |
| BP | GO:1900044 | regulation of protein K63-linked ubiquitination | 13/18862 | 0.012339 | 0.079591 | 0.049178 | SASH1 | 1 |
| BP | GO:1904925 | positive regulation of autophagy of mitochondrion in response to mitochondrial depolarization | 13/18862 | 0.012339 | 0.079591 | 0.049178 | HK2 | 1 |
| MF | GO:0005179 | hormone activity | 122/18337 | 5.31E-06 | 0.000319 | 0.000218 | LEP/INHA/SPX/CRH | 4 |
| MF | GO:0051428 | peptide hormone receptor binding | 21/18337 | 0.000189 | 0.005671 | 0.00388 | LEP/CRH | 2 |
| MF | GO:0005184 | neuropeptide hormone activity | 30/18337 | 0.000389 | 0.00779 | 0.00533 | SPX/CRH | 2 |
| MF | GO:0048018 | receptor ligand activity | 486/18337 | 0.00111 | 0.013937 | 0.009536 | LEP/INHA/SPX/CRH | 4 |
| MF | GO:0030546 | signaling receptor activator activity | 492/18337 | 0.001161 | 0.013937 | 0.009536 | LEP/INHA/SPX/CRH | 4 |
