# Supplementary figures and images for: Integrated Analysis Identifies Four Genes as Novel Diagnostic Biomarkers Which Correlate with Immune Infiltration in Preeclampsia
Source: J Immunol Res. 2022 Apr 28;2022:2373694. doi: 10.1155/2022/2373694 (PMC9071854; doi:10.1155/2022/2373694)

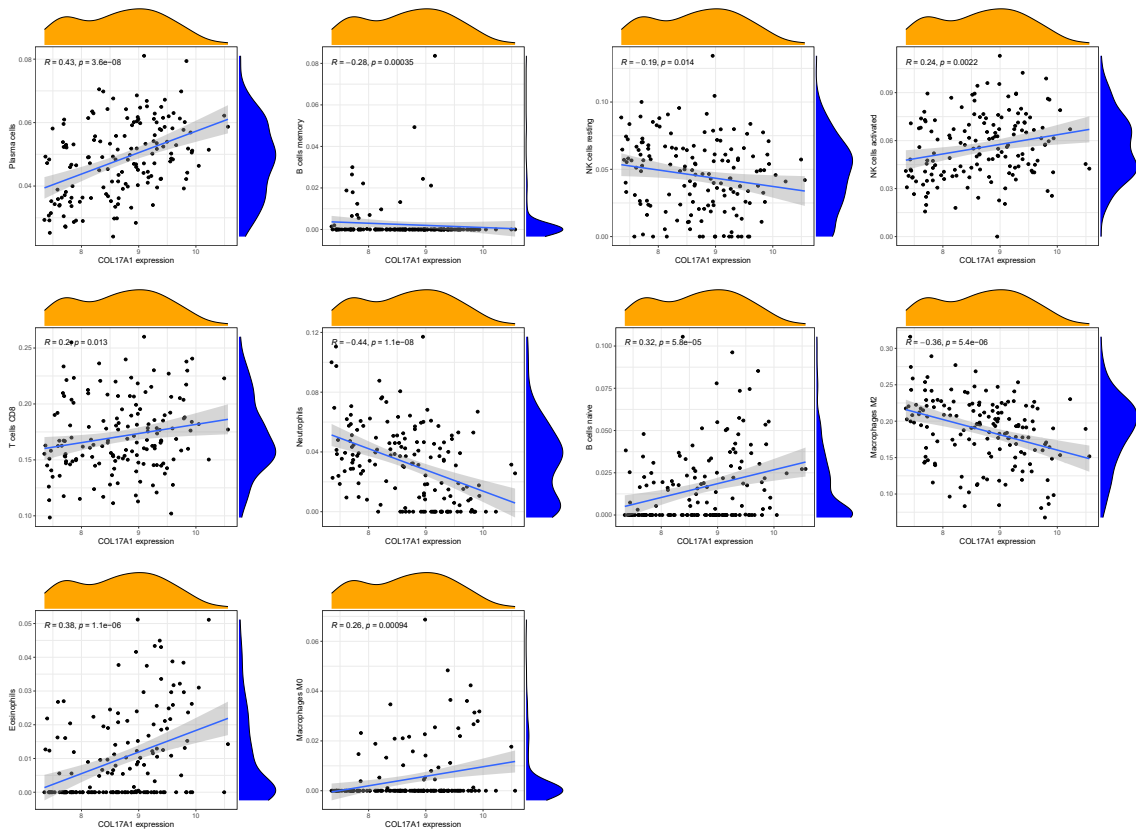

Supplement: Supplementary Materials — Figure S1: correlation of the immune cell percentage with the expression of COL17A1 in preeclampsia. Figure S2: correlation of the immune cell percentage with the expression of FLT1 in preeclampsia. Figure S3: correlation of the immune cell percentage with the expression of FSTL3 in preeclampsia. Figure S4: correlation of the immune cell percentage with the expression of SERPINA3 in preeclampsia. Table S1: the abnormally expressed genes in preeclampsia. Table S2: the details of disease ontology enrichment analysis. Table S3: the details of gene ontology (GO) terms. [file 2373694.f1.zip › Figure S1.pdf]

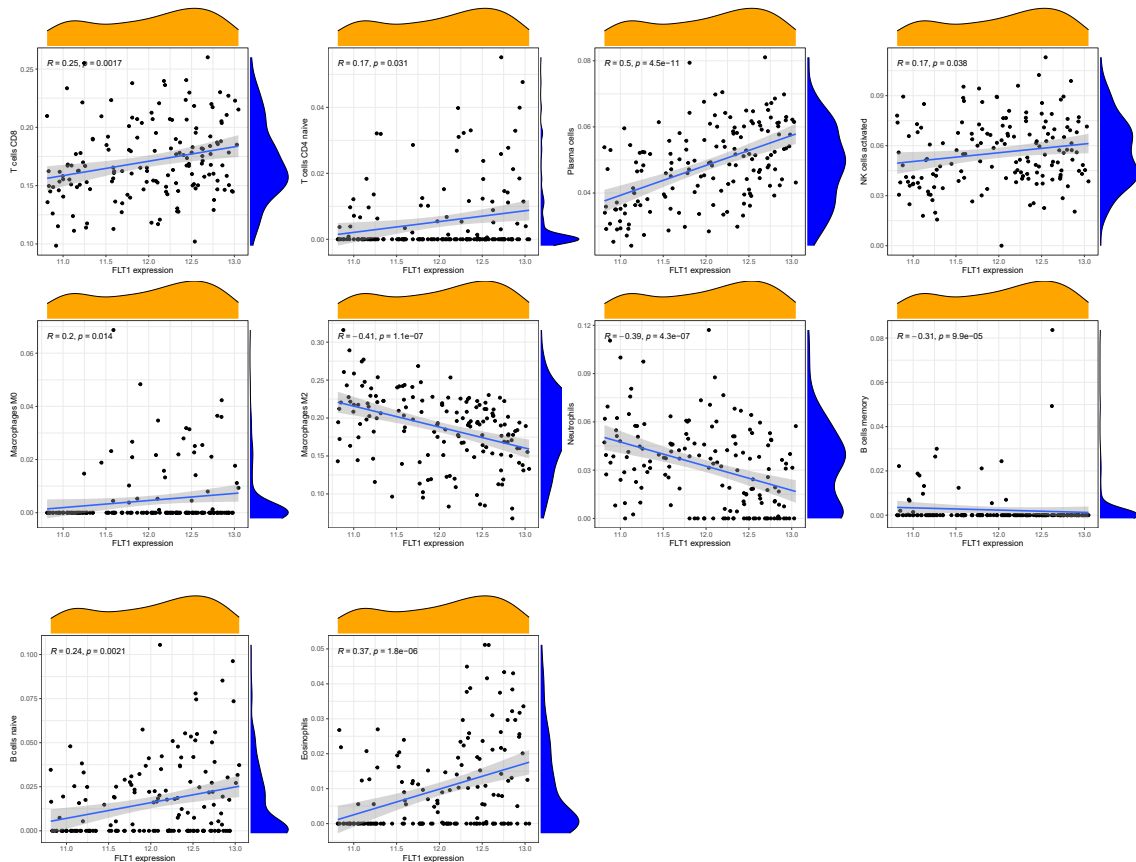

Supplement: Supplementary Materials — Figure S1: correlation of the immune cell percentage with the expression of COL17A1 in preeclampsia. Figure S2: correlation of the immune cell percentage with the expression of FLT1 in preeclampsia. Figure S3: correlation of the immune cell percentage with the expression of FSTL3 in preeclampsia. Figure S4: correlation of the immune cell percentage with the expression of SERPINA3 in preeclampsia. Table S1: the abnormally expressed genes in preeclampsia. Table S2: the details of disease ontology enrichment analysis. Table S3: the details of gene ontology (GO) terms. [file 2373694.f1.zip › Figure S2.pdf]

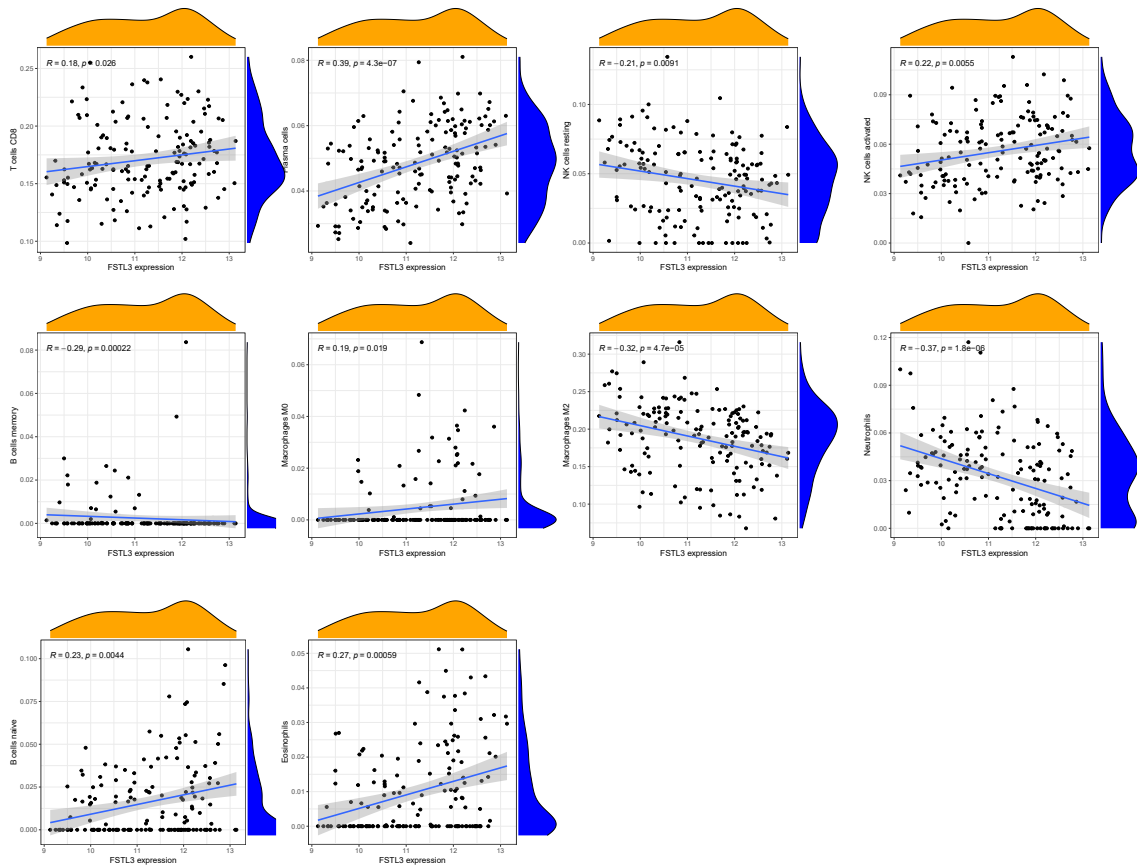

Supplement: Supplementary Materials — Figure S1: correlation of the immune cell percentage with the expression of COL17A1 in preeclampsia. Figure S2: correlation of the immune cell percentage with the expression of FLT1 in preeclampsia. Figure S3: correlation of the immune cell percentage with the expression of FSTL3 in preeclampsia. Figure S4: correlation of the immune cell percentage with the expression of SERPINA3 in preeclampsia. Table S1: the abnormally expressed genes in preeclampsia. Table S2: the details of disease ontology enrichment analysis. Table S3: the details of gene ontology (GO) terms. [file 2373694.f1.zip › Figure S3.pdf]

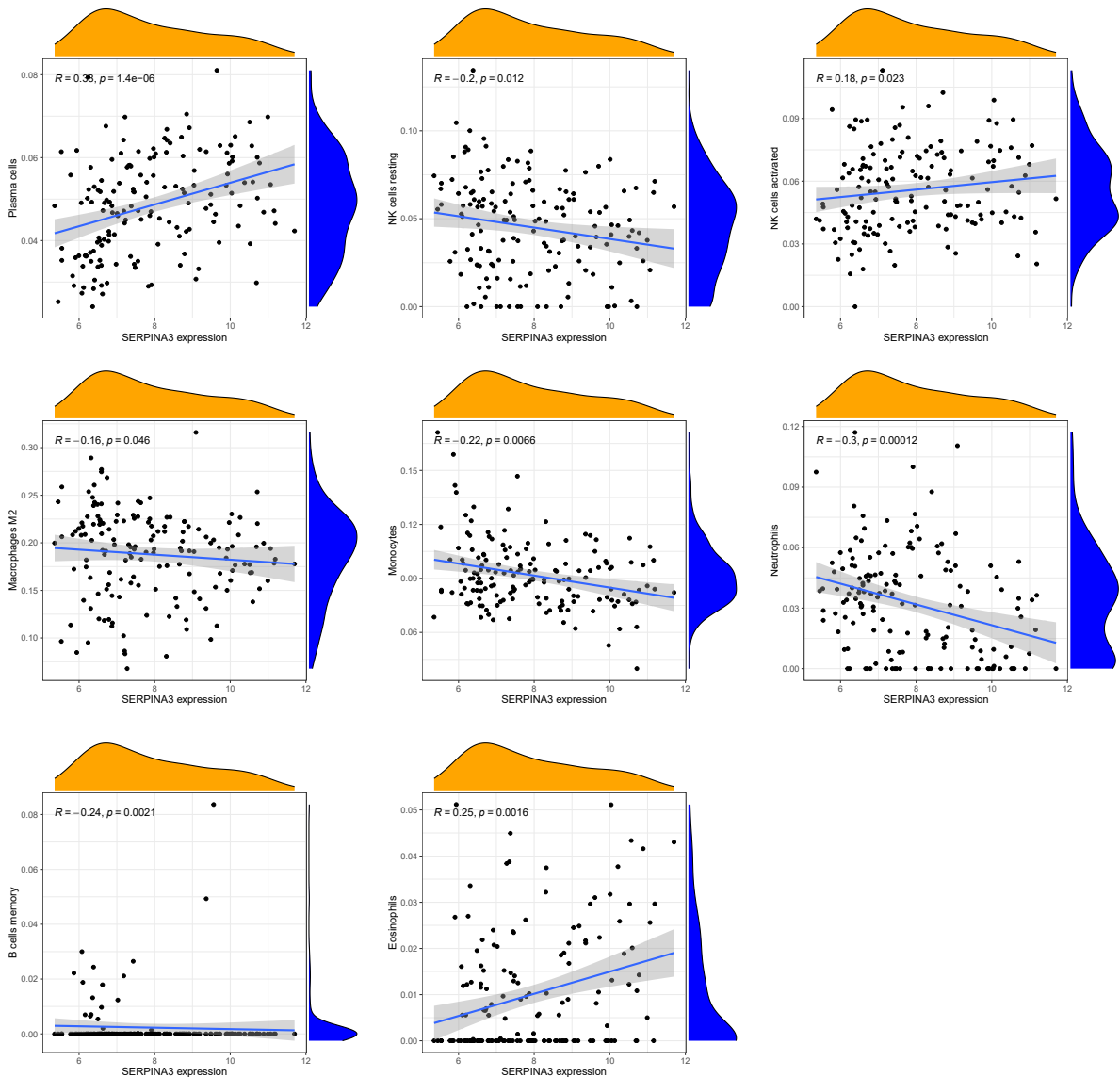

Supplement: Supplementary Materials — Figure S1: correlation of the immune cell percentage with the expression of COL17A1 in preeclampsia. Figure S2: correlation of the immune cell percentage with the expression of FLT1 in preeclampsia. Figure S3: correlation of the immune cell percentage with the expression of FSTL3 in preeclampsia. Figure S4: correlation of the immune cell percentage with the expression of SERPINA3 in preeclampsia. Table S1: the abnormally expressed genes in preeclampsia. Table S2: the details of disease ontology enrichment analysis. Table S3: the details of gene ontology (GO) terms. [file 2373694.f1.zip › Figure S4.pdf]
